# A Growing Role for Gender Analysis in Air Pollution Epidemiology

**DOI:** 10.1289/ehp.0900994

**Published:** 2009-10-16

**Authors:** Jane E. Clougherty

**Affiliations:** Department of Environmental Health, Harvard School of Public Health, Boston, Massachusetts, USA

**Keywords:** air pollution, effect modification, epidemiology, gender, sex

## Abstract

**Objective:**

Epidemiologic studies of air pollution effects on respiratory health report significant modification by sex, although results are not uniform. Importantly, it remains unclear whether modifications are attributable to socially derived gendered exposures, to sex-linked physiological differences, or to some interplay thereof. Gender analysis, which aims to disaggregate social from biological differences between males and females, may help to elucidate these possible sources of effect modification.

**Data sources and data extraction:**

A PubMed literature search was performed in July 2009, using the terms “respiratory” and any of “sex” or “gender” or “men and women” or “boys and girls” and either “PM_2.5_” (particulate matter ≥ 2.5 μm in aerodynamic diameter) or “NO_2_” (nitrogen dioxide). I reviewed the identified studies, and others cited therein, to summarize current evidence of effect modification, with attention to authors’ interpretation of observed differences. Owing to broad differences in exposure mixes, outcomes, and analytic techniques, with few studies examining any given combination thereof, meta-analysis was not deemed appropriate at this time.

**Data synthesis:**

More studies of adults report stronger effects among women, particularly for older persons or where using residential exposure assessment. Studies of children suggest stronger effects among boys in early life and among girls in later childhood.

**Conclusions:**

The qualitative review describes possible sources of difference in air pollution response between women and men, which may vary by life stage, coexposures, hormonal status, or other factors. The sources of observed effect modifications remain unclear, although gender analytic approaches may help to disentangle gender and sex differences in pollution response. A framework for incorporating gender analysis into environmental epidemiology is offered, along with several potentially useful methods from gender analysis.

There is growing epidemiologic evidence of differing associations between air pollution and respiratory health for females and males. More studies report stronger effects among women and girls than among men and boys, but the literature is far from consistent. Importantly, it is unknown whether observed modification is attributable primarily to biological differences between men and women, to exposure differences (e.g., work-related coexposures), or to some interplay thereof. Gender analysis, which aims to disaggregate social and biological differences between men and women (e.g., hormonal status), may help to elucidate this modification, identify key mechanisms, and design more effective interventions.

The distinction between gender (i.e., self-representation, socially derived activities and roles) and sex (i.e., biological differences by chromosomal complement, including reproductive organs and hormonal composition) (Krieger 2003) speaks to the distinction between exposure and susceptibility. Gender analysis is more common in occupational epidemiology (Arbuckle 2006; Messing and Stellman 2006; Messing et al. 2003; Schachter et al. 2009) than in environmental health (Keitt et al. 2004), because persistent job stratification by sex (Alexanderson and Östlin 2001) has produced marked differences in occupational exposures to chemical agents (Hursidic-Radulovic et al. 2002; London et al. 2002), ergonomic demands (Silverstein et al. 1986), injury (Salminen et al. 1992), and psychosocial stressors (Arcand et al. 2000; Bourbonnais et al. 2000; Gutek 2001; Hall 1989).

Gender, a social construct, includes cultural norms, roles, and behaviors shaped by relations among women and men and among girls and boys (Krieger 2003). Gender, inherently social, varies continuously over multiple dimensions over the life course, whereas sex is normally dichotomous. Gender is shaped at the societal level and varies across nation, culture, class, race, ethnicity, nationality, sexuality, and religion. Gender describes patterns of behavior, place, and role, determining where people spend time and their activities, thereby shaping exposure distributions.

Sex, a biological construct, is based on physiologic differences enabling reproduction, defined by physiologic characteristics (especially reproductive organs) or chromosomal complement (Krieger 2003). Sex-linked traits (e.g., hormonal status, body size) influence biological transport of environmentally derived chemicals. Lung size and growth, deposition of fine particles [particulate matter ≥ 2.5 μm in aerodynamic diameter (PM_2.5_)] (Kim and Hu 1998, 2006), gas absorption (Jones and Lam 2006), gas–blood barrier permeability (Brauner et al. 2009), airway hyperresponsiveness (Kanner et al. 1994), vascular response (Prisby et al. 2008), and inflammation (Hermes et al. 2006; Sood et al. 2008) all differ, on average, by sex.

Sex and gender can be difficult to distinguish in epidemiologic data; they are tightly intertwined, with reciprocal effects. Biological characteristics (e.g., body size) become engendered as occupational and family roles, which are gendered expressions of biology. Likewise, gendered work and caregiving roles, smoking, and alcohol consumption influence muscle mass, adiposity, and chemical body burden—collectively, these are socially derived biological expressions of gender (Krieger 2003).

In this review I present a framework for incorporating gender analysis into air pollution epidemiology, describing pathways through which gender and sex, separately and multiplicatively, may influence pollution response. Current evidence of effect modification in air pollution respiratory epidemiology is summarized, and potentially useful nascent analytic methods from gender analysis are offered.

Gender analysis explores topics far beyond those addressed here, including sexuality and transgender issues. Here I consider only those constructs and tools that may directly inform mean differences between men and women in air pollution epidemiology.

## A Framework for Incorporating Gender Analysis into Environmental Epidemiology

**Figure 1:**
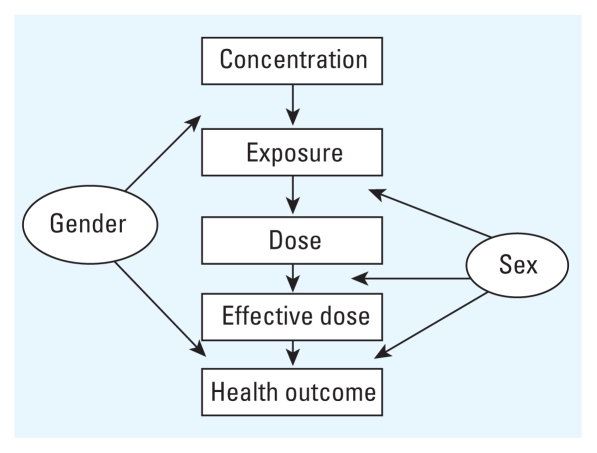
Possible roles of gender and sex in shaping observed relationships between air pollution and health. Gender affects the presence of the exposure itself (e.g., cosmetic use), whereas biological sex differences determine the consequent dose (e.g., through dermal thickness and permeability). Sex differences in biological transport and target organs determine health outcomes, potentially modified by gendered (behavioral) coexposures and their sequelae.

Incorporating aspects of gender analysis into the environmental health paradigm (Figure 1) actualizes this distinction between gender and sex. The framework is elucidated by drawing examples broadly from environmental epidemiology, elucidating pathways through which gender and sex may, individually and recursively, shape population exposure and susceptibility.

### Concentration to exposure

Gender shapes where people spend time and activity patterns—for example, sports participation, work-related chemical and ergonomic exposures, and use of personal care and cleaning products. Nickel dermatitis and hand eczema are far more prevalent among women than men in Western countries, likely because of chronic exposures from jewelry (Meding 2000). Indoor fossil fuel burning for cooking in developing countries drastically increases kitchen PM_2.5_ concentrations (Dionisio et al. 2008; Rumchev et al. 2007); because women generally perform more cooking in these societies, they suffer elevated respiratory symptoms (Behera 1997), asthma (Qureshi 1994), chronic bronchitis (Pandey 1984), chronic obstructive pulmonary disease (COPD) (Ramirez-Venegas et al. 2006), pneumoconiosis (Grobbelaar and Bateman 1991), tuberculosis, lung cancer (Behera and Balamugesh 2005), and mortality (Lopez et al. 2006). Accordingly, stove-replacement interventions have effectively reduced exposures and improved women’s health in these settings (Khushk et al. 2005; McCracken et al. 2007). Gendered home activities shape exposures to cooking exhaust and cleaning products, behaviors and home characteristics that vary by social class, climate, and culture. Residence-based exposure estimates may better capture exposures among homemakers and thus may be more accurate for women than men in most societies.

### Exposure to dose

Sex differences in dermal absorption and lung function (Becklake and Kauffmann 1999; Ernstgard et al. 2002) influence contaminant uptake. Skin metabolizes some xenobiotics, modifying their toxicity (Bashir and Maibach 2002); this characteristic differs by sex and is influenced by gendered dermal exposures (e.g., topical creams, cosmetics, jewelry). Respiratory absorption of airborne gases (Jones and Lam 2006) and gas–blood barrier permeability (Brauner et al. 2009) also differ by sex.

### Dose to effective dose

Sex determines the availability of target organs and hormonal systemic regulation. Only in women are patterns in ovarian cancer or pregnancy outcomes observable; only in men can testicular cancer patterns be observed. Kinetics and toxicity of chemicals in women’s bodies vary across the life course, during menarche, pregnancy, lactation, and menopause (Roberts and Silbergeld 1995; Silbergeld et al. 1988); gastrointestinal cadmium accumulation increases with low iron stores (Åkesson et al. 2002), common during pregnancy and among women of reproductive age (Gunshin et al. 1997). Estradiol and testosterone influence transport of environmentally derived chemicals and accumulation in the brain, kidney, liver, and intestines (Morris et al. 2003); mercury retention in kidneys can be three times higher among women than men (Barregård et al. 1999; Hultman and Nielsen 2001). During pregnancy (a sex-linked state), activity and exposure patterns change (Nethery et al. 2009), and hormonal changes affect toxicant transport throughout the body.

### Effective dose to health outcome

Sex-linked biological differences influence disease etiology after organ exposure. Women have more arsenic-induced kidney and bladder cancers than do men in regions with arsenic in drinking water, likely because of reduced chemical excretion during pregnancy and lactation (Concha et al. 1998). Sex-linked hormonal status alters vascular effects of diesel exhaust (Prisby et al. 2008). Coexposures from gendered behaviors (e.g., alcohol and tobacco use, cardiovascular exercise) modify the biological fate of environmentally derived chemicals and organ resiliency. Sex and gender effects can interact; sex-linked pregnancy outcomes (observable only among women) are modified by gendered behaviors (e.g., smoking, occupational endocrine disruptors, hairspray exposures) (Ormond et al. 2008). Gender differences in health-care seeking and illness behaviors influence the progression of environmentally derived illness.

## Current Evidence of Effect Modification by Sex in Air Pollution Epidemiology

### Search Methods

A PubMed (National Library of Medicine 2009) search, performed in July 2009, retrieved all publications in the database identifiable using the terms “respiratory” and “nitrogen dioxide” (or “NO_2_”) and any of the following terms: “sex” (*n* = 41 citations), “gender” (*n* = 8), “women and men” (or “men and women”) (*n* = 243), or “girls and boys” (or vice versa) (*n* = 8). Another search retrieved all publications identifiable using “fine particulate matter” (“PM_2.5_”) and “respiratory” and any of the following terms: “sex” (*n* = 11), “gender” (*n* = 5), “women and men” (or vice versa) (*n* = 65), or “girls and boys” (or vice versa) (*n* = 2). Only respiratory outcomes were considered (i.e., diagnosed respiratory illness, symptoms, lung function, respiratory mortality), although the findings and models may apply to other outcomes. Papers examining noninhalation pathways were also excluded; thus, effects of prenatal air pollution exposures on infant and child health (which may differentially affect boys) are not considered here.

Of the 383 publications identified, seven review articles were eliminated, along with 30 duplicate citations identified by multiple search criteria, 42 publications not available in English, 50 publications on noninhalation pathways or nonrespiratory outcomes, 13 publications on nonhuman species, and 32 publications not primarily examining air pollution exposures. Abstracts of the remaining 209 publications were reviewed to determine whether effect modification by sex was tested; if the abstract was unclear, the original publication was consulted.

Most publications reported only sex-adjusted effects or examined only one sex. Only 37 unique publications examined air pollution effect modification by sex (summarized in Tables 1 and 2). Given vast differences in analytic methods, outcomes, exposure intensities, and durations—with few studies exploring any combination thereof—meta-analysis was not appropriate. It is beyond the scope of this review to assess the magnitude of effect modification, which varies by study design and outcome measure. Most (not all) of the reviewed publications reported odds ratios or risk ratios, with interactions on the multiplicative scale. Authors also used varying statistical criteria for “significant” interactions (here, *p* < 0.05 unless otherwise stated). Issues in assessment of interactions for epidemiology have been detailed elsewhere (Knol et al. 2009).

The qualitative review documents the widely varying explanations offered to explain observed modifications—as such, only papers in which authors offered such interpretations are included. Accordingly, the results described here, and summarized in Tables 1 and 2, are not exhaustive, but represent effect modification as reported by the authors. Only a few studies took additional analytic steps to examine sources of difference that may account for observed effect modification.

### Search Results

Because gender differences in behaviors, exposures, or coexposures (e.g., diet, smoking) and biological factors (e.g., hormonal composition) change over the life course, studies are summarized separately for adults and children.

#### Gender and sex differences in respiratory health effects among adults

##### Studies reporting stronger effects among women

Studies of residential air pollution exposures suggest stronger associations among women. In the Atherosclerosis Risk in Communities (ARIC) study, Kan et al. (2007) found that living near a major road predicted lower forced expiratory volume in 1 sec (FEV_1_) and forced vital capacity (FVC) only among women. The authors pointed to women’s greater airway reactivity, citing stronger responses to smoking (Lodrup Carlsen et al. 2006; Vollmer et al. 1994; Yunginger et al. 1992), or better accuracy in residential exposure assessment for homemakers (35% of ARIC women vs. 17% of men).

Franklin et al. (2007) studied 130,000 respiratory deaths in 27 U.S. communities, using case–crossover methods and meta-analysis, and found that community air pollution better predicted death among women than among men. The authors proposed sex-differing respiratory anatomy and physiology, or PM deposition patterns.

In a comprehensive study of daily air pollution and respiratory hospitalization among adults and children in Windsor, Ontario, using time-series and case–crossover methods, Luginaah et al. (2005) reported a larger number of significant associations among women and girls than among men and boys. Two-day lagged coefficient of haze (COH) exposures predicted increased risks among women. For girls 0–14 years of age, 1- to 2-day lagged NO_2_, sulfur dioxide (SO_2_), and carbon monoxide (CO) exposures predicted elevated risks. Among males, only 1-day lagged PM_10_ predicted increased risks among adults. The authors proposed sex-differing biological explanations (e.g., hormonally affected inflammation, smooth muscle and vascular function, lung growth and decline, airway and parenchymal size), citing evidence of sex-differing airway PM_2.5_ deposition (Kim and Hu 1998; Kohlhaufl et al. 1999) and greater responsivity to tobacco smoke among females (Chen et al. 1999, 2005; Gold et al. 1996; Hursidic-Radulovic et al. 2002; Prescott et al. 1997; Varkev 2004; Xu et al. 1994a, 1994b). They considered gendered explanations; women are, on average, poorer and may experience greater (or different) psychosocial stressors, perform more household tasks (increasing exposures to viral infection, indoor allergens, combustion exhaust, cleaning solvent, and aeroallergens) (Redline and Gold 1994), and may differ from men in health-care seeking and illness management behaviors (Goodman et al. 1994).

One Chicago cohort studied by Ito and Thurston (1996) showed greater all-cause and respiratory mortality with same- and previous-day PM_10_ among black women than among other sex/race groups. The authors observed that physiologic differences and gender differences in activities, occupation, and class may shape pollution response, noting that race and gender were yet unexplored in environmental epidemiology.

In the Public Health and Air Pollution in Asia (PAPA) study, Kan et al. (2008) reported stronger associations between pollutants [PM_10_ (PM with aerodynamic diameter < 10 μm) SO_2_, NO_2_, ozone (O_3_)] and daily respiratory mortality among women, elderly, and lower socioeconomic status (SES) persons. The authors offered gendered explanations (e.g., smoking among men may obscure pollution effects; Shanghai women’s lower average education may confound gender and SES) and considered biological explanations, including women’s smaller airways, greater airway reactivity (Yunginger et al. 1992), and greater deposition of PM_2.5_ (Kohlhaufl et al. 1999; Sunyer et al. 2000).

Among 6,824 adults in 10 European countries in the European Community Health Survey 2000–2002 (ECRHS I), Sunyer et al. (2006) found that home traffic intensity and outdoor NO_2_ better predicted chronic bronchitis among women than among men. The authors also examined occupational exposures, which better predicted outcomes among men, separating some gendered activity pattern effects. The authors suggested sex-linked differences in hormonal status, and gender differences in coexposures, disease perception, health care access and use and differing perceptions of environmental quality and symptoms by gender and education.

Sunyer et al. (2000) found that older and female Barcelona adults with COPD showed greater all-cause, respiratory, and cardiovascular mortality with same-day black smoke than did younger persons and men. The authors suggested the reasons were a higher prevalence of frail persons among the elderly and women than among men, or biological differences, including inflammatory response [given women’s stronger response to smoking (Xu et al. 1994a, 1994b)], lung size, and airway diameter influencing PM deposition, respiratory patterns, and airway resistance (Bennett et al. 1996).

##### Studies reporting stronger effects among men

In the 20-year prospective California Adventists Health Study, Abbey et al. (1998) linked PM_10_ to reduced lung function (FEV_1_/FVC) among nonsmoking males, and decreased FEV_1_ among men with parental respiratory illness. Women and never-smoking males displayed increased peak expiratory flow (PEF) lability. Among males, sulfate exposures predicted reduced FEV_1_, and O_3_ exposures predicted reduced FEV_1_ among men with parental respiratory illness. The authors suggested gender differences in work-related exposures or possible stronger healthy worker effects among women. They confirmed that cohort men spent more time outdoors (16.1 hr/week vs. 9.2 hr/week; *p* < 0.0005) and suggested that outdoor exposures may trigger responses in males with genetic predisposition to respiratory illness.

Galizia and Kinney (1999) found that, among Yale freshmen, growing up in areas with high (vs. low) O_3_ was associated with symptoms and reduced lung function among males but not among females. The authors suggested the gendered explanation that men may accumulate greater O_3_ exposures through outdoor physical activity.

##### Studies reporting null or mixed modification

Zeka et al. (2006) found that ambient PM_10_ was associated with respiratory and all-cause mortality across 20 U.S. cities, using case–crossover analysis. Although modification was nonsignificant, the authors posited that sex, race, and age may indicate SES, increasing susceptibility through lesser health care access, poorer nutrition, greater stress or violence exposures, or increasing actual exposures through residential proximity to highways or occupational coexposures. Finally, they suggest sex-linked biological differences in PM deposition.

In a 13-year follow-up of Krakow adults, Jedrychowski and Krzyzanowski (1989) found that residence in higher sulfate areas better predicted FEV_1_ decrements among men than among women. Among women, SO_2_ and PM correlated with symptoms; the authors suggested that women’s greater average spent time near home produced better accuracy in exposure assessment.

#### Gender and sex differences in respiratory health effects among children

Disentangling gender and sex effects in air pollution–health associations among children may be more complicated, because lung function growth rates (critical periods for pollution effects) differ by sex (Berhane et al. 2000). Most air pollution epidemiology studies among children examine chronic exposures, although outcomes considered vary widely, including lung function growth, wheeze, asthma onset and exacerbation, and symptoms.

##### Studies reporting stronger effects among girls

Using baseline cross-sectional results from the Southern California Children’s Health Study (CHS) of children in grades 4, 7, and 10 in 12 communities, Peters et al. (1999) reported that air pollutants (PM_10_, PM_2.5_, acid vapor, NO_2_, O_3_) were more strongly inversely associated with lung function among girls than among boys. The authors suggested gender differences in time outdoors and play activities, and sex differences in growth rates, hormonal factors, and respiratory mechanisms. Using longitudinal CHS analyses, Gauderman et al. (2004) found deficits in FEV_1_ growth from 10 to 18 years of age associated with community NO_2_, PM_2.5_, and acid vapor not significantly differing by sex. McConnell et al. (2002) reported higher asthma risk with outdoor sports participation in higher O_3_ areas in the CHS cohort, especially among girls, and suggested that higher ventilation during play may increase exposures.

In a U.S. study, Neas et al. (1991) reported stronger associations between home indoor NO_2_ and respiratory symptoms among girls than among boys 7–11 years of age. The authors cited reports of stronger effects among girls, including a British study linking gas stove use to symptoms among girls (Melia et al. 1977), a paper reporting FEV_75_ (75th percentile) decrements of 1.1% among girls 9–13 years of age but slight increases among boys (Hasselblad et al. 1981), and a British study linking kitchen NO_2_ and gas stoves to greater reductions in PEF and forced expiratory flow between 25th and 75th percentile (FEF_25–75_) among girls (Florey et al. 1979).

Among Dutch children 7–12 years of age, Brunekreef et al. (1997) found that truck traffic and black smoke at schools were associated with lung function reductions only among girls, and van Vliet et al. (1997) found that residential distance from freeway, truck traffic density, and school black smoke measures better predicted chronic respiratory symptoms among girls than among boys, after accounting for SES and home exposures. In both studies, the authors contrast their results with evidence of stronger passive smoke effects among boys. However, these studies examine *in utero* exposures and noninhalation pathways (Cuijpers et al. 1995; Cunningham et al. 1994), and suggest that, because boys exhibit more symptoms overall, air pollution effects may be obscured by other respiratory “noise” (Pershagen et al. 1995).

Among 673 adults and 106 children in Haarlem, the Netherlands, Oosterlee et al. (1996) reported significant associations between living along busy (vs. quiet) streets and asthma or dyspnea only among girls. They suggested that boys’ higher total respiratory symptoms may mask pollution effects, and considered gendered factors (e.g., passive smoking, activity patterns, coexposures) in their analysis.

In Oslo, Norway, Oftedal et al. (2008) found that lifetime residential NO_2_, PM_10_, and PM_2.5_ among 9- and 10-year-old children was associated with lower PEF, more strongly among girls, only slightly attenuated by SES adjustment. The authors suggested biological explanations (e.g., girls experience growth spurts earlier, captured within this follow-up, or hormonal status may alter girls’ responses) and suggested unmeasured SES-related confounders (e.g., gendered sports participation).

In a case–control study in Stockholm, Pershagen et al. (1995) reported significant associations between outdoor home NO_2_ and gas stove use on wheezing bronchitis only among girls, despite boys’ higher wheezing prevalence. Outdoor NO_2_, gas stove use, and smoking conferred multiplicative risks in girls but not in boys, after SES adjustment. The authors reported consistency with prior studies, indicated that results were unlikely due to selection bias or misclassification, and acknowledged a need for activity data to explore gender differences.

Rosenlund et al. (2009) found associations between chronic residential NO_2_ exposure and lung function to be stronger among Roman girls than boys 9–14 years of age; mean FEV_1_ and FEF_25–75_ decrements were approximately four times greater in girls than boys, corroborating other studies (Cesaroni et al. 2008; Kulkarni et al. 2006; Li et al. 2003; Neas et al. 1991; Nordling et al. 2008; Oftedal et al. 2008; Pershagen et al. 1995). The authors indicated complexities in comparing childhood cohorts differing by age, pubertal status, pollution mixtures, study designs, and susceptibilities and noted that the consistency of results across Europe reporting stronger air pollution effects among girls, meriting further investigation.

##### Studies reporting stronger effects among boys

In the Traffic-Related Air Pollution on Childhood Asthma (TRAPCA) study, Gehring et al. (2002) reported stronger associations between residential PM_2.5_ and symptoms (e.g., cough without infection, cough at night) among boys than among girls 0–2 years of age. The authors suggested that differences in total symptoms, masking pollution effects, were important or that, given sex differences in lung development, infant girls have larger airways relative to body size and lesser airway resistance.

In a prospective cohort study of annual mean total suspended particle (TSP) and SO_2_ exposures among preadolescent children in Krakow, Poland, Jedrychowski et al. (1999) reported stronger associations with FVC and FEV_1_ among boys than among girls. The authors noted sex-differing lung growth rates, producing different critical periods for pollution effects.

##### Studies reporting null or mixed effect modification

In a 3-year prospective study of children in Mexico City, Mexico, Rojas-Martinez et al. (2007) associated elevated PM_10_, NO_2_, and O_3_ with reduced lung function among boys and girls. Interquartile range increases in NO_2_ predicted FEV_1_ declines in girls, whereas increases in PM_10_ predicted FEV_1_ declines among boys. Elevated O_3_ predicted FEV_1_ decreases three times larger among girls than among boys, unexplained by SES. The authors compared these findings with CHS results on sex-differing lung function growth and suggested higher O_3_ exposures among children spending time outdoors (Gauderman et al. 2000; McConnell et al. 2002).

In Toronto (Ontario, Canada), respiratory hospitalizations were significantly associated with PM_2.5–10_ among boys and girls, with PM_10_ among boys, and with NO_2_ among girls (Lin et al. 2005). The authors proposed sex-linked explanations: Boys have smaller airways relative to lung volume and differ in smooth muscle, vascular function, and hormonal status.

## Discussion

Among adults, evidence of effect modification by sex remains uncertain; studies of older adults and those using residential exposure estimates suggest stronger effects among women. The range of plausible explanations is very broad, including sex-linked biological factors related to lung volume, deposition, reactivity, and hormonal influences on chemical transport and systemic regulation. Gendered explanations include confounding or modification by smoking behaviors, job-related chemical exposures, differential accuracy in residence-based exposure assignment, exposures to indoor allergens and cleaning agents, and differing exposure and response to psychosocial stressors. Refined distinction between sex and gender may elucidate these associations.

Studies of younger children suggest stronger associations among boys; older childhood cohorts suggest the opposite. Age-related trends may be linked to sex-differing lung function growth rates (Gold et al. 1994) and differences in airway function at birth, which suggest lower respiratory volumes and greater airway resistance among boys (Stocks et al. 1997). At older ages, gendered activities may also shape pollution response.

### Gender, sex, and multiple exposures

Environmental exposures are complex. Traffic-related air pollution includes gaseous species and PM from combustion, tire and brake wear, resuspended roadway dusts, and salts (Schauer et al. 2006). Pollution exposures occur in multiplicity, and polluted neighborhoods often also suffer poverty, crime, and lower access to health-related resources (Morello-Frosch and Shenassa 2006). In workplaces, chemical exposures co-vary with heat, noise, and strain, acting recursively and synergistically on workers’ health (MacDonald et al. 2001). Gender analysis fits into environmental health under this multiple exposures framework. There is growing interest in pollution effect modification by SES (Jerrett et al. 2004; O’Neill et al. 2003) and chronic stress (Chen et al. 2008; Clougherty et al. 2006, 2007; Morello-Frosch and Shenassa 2006). Likewise, SES is a complex mix of social and physical stressors accumulating over the life course (Romieu et al. 2005), shaping health and susceptibility. Behavioral and physiologic responses to SES and stressors may vary by gender (Seeman et al. 2002); women, on average, may respond more strongly to interpersonal stressors (Davis et al. 1999) and experience different physiologic sequelae (Hermes et al. 2006; Iwasaki-Sekino et al. 2009; Seeman et al. 1995). Women’s behavioral responses may emphasize social support, caregiving, and child tending (Taylor et al. 2000), whereas better known “fight-or-flight” responses emphasize sympathetic–adrenal–medullary enervation and activities linked to traditionally male roles (Powell and Matthews 2002; Taylor et al. 2000). Stress may be a gendered factor (i.e., exposures differ by gender) and a sex-differing factor as well, if physiologic responses to stress differ (e.g., sex-differing epinephrine responses). If stress modifies pollution response, then understanding gendered stress responses is likely important for accurately characterizing gendered pollution responses.

Research from social geography may help to better elucidate gendered spatial and behavioral exposure patterns. Gendered use of space and exposure patterns in urban communities is evident in the example of fear of violence. One large U.S. survey reported that 26% of women “never” leave home after dark (vs. 9% of men), 51% “always” bring friends for protection (vs. 4% of men), and 71% consider safety when parking (vs. 33% of men) (Gordon and Riger 1991). Strong gender differences in perceived safety shape activity and exercise patterns; parents’ greater restriction of girls’ geographic range in U.S. cities shapes exposure paradigms, exercise, experience, and developmental opportunity (Katz 1993). Better understanding the gendered environment can improve exposure assessment, better isolate biological responses, and provide a model for examining other social effect modifiers (Clougherty and Kubzansky 2009).

### Analytic approaches for disentangling effects of gender and sex

Because gender and sex are tightly intertwined, their effects can be difficult to distinguish in epidemiologic data. “Gender” and “sex” have commonly been conflated in epidemiologic research (Krieger 2003). Most important, careful use of language distinguishing these constructs will enable researchers to better describe and understand sources of difference in exposure–health relationships. Methodology for gender analysis is an evolving field, although the methods described here may help to disentangle some effects of sex and gender and may merit further exploration in environmental epidemiology.

Reporting sex-stratified results is more informative than is adjustment for sex (Arbuckle 2006) and can identify associations differing broadly between males and females. However, sex stratification often confounds tightly correlated gender and sex effects, obscuring true sources of difference. Preferably, researchers may stratify data separately by multiple sex- and gender-associated factors (e.g., body size, working outside the home, time spent on household tasks) to elucidate sources of difference. Most epidemiologic data sets are not adequately powered to perform multiple stratifications simultaneously, so these multiple stratifications usually need be performed separately. Stratification variables should reflect time–activity patterns or meaningful biological factors, rather than stereotypical attributes, to identify true factors relevant to the cohort under study.

Population-specific exposure modeling may improve culturally and behaviorally specific exposure assessment, clarifying gendered exposure differences. Residential exposure metrics may be more accurate for women, who spend more time near home on average, especially when caring for children or other family members (Gilliand et al. 2005; Kingsley 2003; Maziak et al. 2005; Payne-Sturges et al. 2004). Residential activities may require microenvironmental exposure assessment (LaRosa et al. 2002), because gendered activities (e.g., cooking, cleaning, lawn care) produce different exposure patterns. Exposure measurement may benefit from gendered exposure measurement, comparison of gendered activities across communities (Berhane et al. 2004), or foci on temporal exposure characteristics (e.g., diurnal trends in residential exposures and activities, critical life-course periods related to hormonal composition or roles) (Gilliand et al. 2005). Assignment of gendered exposures broadly to sex-stratified groups, however, should be generally avoided, because this practice obscures sources of variability between men and women, further confounding sex effects in subsequent epidemiologic analyses.

Temporally refined exposure assessment may elucidate gendered activity distributions. Recent approaches include probabilistic modeling of personal exposures (Zidek et al. 2005). Techniques from the social sciences may be useful; the experience sampling method (Csikszentmihalyi and Larson 1987) uses cell phones or pagers to prompt individuals throughout the day to record their location, activities, and well-being. The technique improves upon diary entries, which suffer recall bias, and allows more detail in activity reports (e.g., cleaning activity with duration and product name) with contemporaneous physiological or psychological conditions that may modify effects. Aggregated, the data provide population-specific activity distributions and capture mean daily activity and exposure differences between men and women.

Physiologically based pharmacokinetic (PBPK) modeling may help to distinguish sex differences in dermal absorption, body size, and toxicity (Arbuckle 2006; Meibohm et al. 2002) from gendered exposures. PBPK models may facilitate analysis of biological processes across multiple life stages (e.g., infancy, childhood, puberty, adulthood) and, among women, by reproductive cycle and hormonal status (e.g., menarche, pregnancy, lactation, menopause). Better understanding of sex and life-stage aspects of bodily chemical transport may help to elucidate differences in effective dose or chemical interactions in the body.

Propensity analysis incorporates predictive modeling for both exposures and responses, enabling researchers to predict subjects’ propensity (likelihood) of exposure, given preexposure characteristics and population exposure distributions. Researchers can then examine health responses among individuals with comparable exposure likelihoods, using propensity matching or propensity stratification (Kurth et al. 2006). For example, sex-stratified propensity models can estimate effects of education, work history, SES, family structure, and home demands on exposure assignment (e.g., job, neighborhood of residence) for men and women. Then researchers can better observe health responses by sex, reasonably isolating effects of mean biological differences from those of gendered exposure assignment. One recent occupational study examined blue-collar status and hypertension among employees of a large U.S. manufacturing company (Clougherty et al. 2009). Family structure influenced exposure (job) assignment for men and women; single mothers were more likely to be blue-collar workers than were other women. Men with partners and children were more likely to be white-collar workers than were other men. Blue-collar status increased risks solely among women predicted to be blue collar, suggesting interaction effects between SES (which predicted job assignment) and on-the-job exposures.

Finally, researchers have proposed variants of multilevel modeling (Phillips 2005) to disaggregate variability between and within the sexes. Researchers may differentiate sex-linked biological effects (e.g., target organs, hormonal composition), which can differ substantially between men and women, from gendered exposures, which generally display more variability among men and women. The technique may be applicable, however, only to illnesses directly involving biological parameters (e.g., sex organs, hormonal composition) which differ strongly by sex. A different method for employing multilevel modeling stems from the societal-level construction of gender, whereas sex is an individual-level biological construct. Examining men’s and women’s exposure and disease patterns across and within societies that vary in measures of gender equity (e.g., income disparities, female education, reproductive rights) may offer important clues toward understanding root causes of exposure and susceptibility differences (Phillips 2008).

## Conclusions

Studies suggest that health responses to air pollution may differ between women and men and between girls and boys. It remains unclear, however, whether observed modification is a result of sex-linked biological differences (e.g., hormonal complement, body size) or gender differences in activity patterns, coexposures, or exposure measurement accuracy. Most modification likely consists of some combination of these two factors (exposure patterns and biological response); disentangling these effects is challenging yet necessary toward fully understanding the relevant pathways for differential air pollution effects on health.

Because gender varies by state and society, designing effective localized health interventions requires clarity about these distinct sources of difference (gender and sex), with an aim of improving population health. Careful consideration of gender and sex effects and exploration of nascent methods for quantitative gender analysis may help to elucidate sources of difference. More broadly, exploring the role for gender analysis in environmental epidemiology may provide a model for exploring other social factors that can shape population responses to air pollution.

## Figures and Tables

**Table 1:** Studies examining effect modification by sex among adults.

Study	Population	Exposure metric(s)	Outcome(s)	Risk among males	Risk among females
Studies reporting stronger effects among women					
Franklin et al. 2007	1.3 million deaths, 27 U.S. cities 1997–2002	Prior day PM_2.5_ > 10 μg/m^3^	Percent increase in respiratory mortality All-cause mortality	1.90 (0.14–3.65) 1.06% (0.07–2.6)	1.57% (−0.22 to −3.35) 1.34% (0.40–2.27)

Ito and Thurston 1996	Daily deaths in Chicago, IL 1985–1990	Daily PM_10_, O_3_ at nearest regulatory monitor	RR for respiratory mortality	RR = 1.10 (0.97–1.26)	RR = 1.17 (1.02–1.35)

Kan et al. 2007	15,792 middle-age U.S. adults, 1987–1989 (ARIC cohort)	Quartiles of residential traffic density	Lung function: FEV_1_ FVC	β (Q4, age adjusted) = 19.6 (−34.9 to 74.1); *p*-trend = 0.66 β (Q4, multivariate) = 11.7 (−40.2 to 63.5); *p*-trend = 0.86	β (Q4, age adjusted) = −34.8 (−66.5 to −3.1); *p*-trend = 0.01 β (Q4, multivariate) = −34.8 (−66.5 to −3.1); *p*-trend = 0.01

Kan et al. 2008	Adult population of Shanghai, China (population, 13.1 million)	10-μg/m^3^ increase in daily PM_10_, SO_2_, NO_2_, O_3_	Percent increase in respiratory mortality	β (PM_10_) = 0.17% (0.03 to 0.32) β (SO_2_) = 0.85% (0.43 to 1.28) β (NO_2_) = 0.88% (0.49 to 1.28) β (O_3_) = 0.19% (−0.16 to 0.55)	β (PM_10_) = 0.33% (0.18–0.48) β (SO_2_) = 1.06% (0.62–1.51) β (NO_2_) = 1.10% (0.69–1.51) β (O_3_) = 0.40% (0.03–0.76)

Luginaah et al. 2005	1,602 adults (15–64 years) in Windsor, Ontario, Canada 1995–2000	IQR increase in 1-, 2-, 3-day lag NO_2_, SO_2_, CO, COH, O_3_, PM_10_, TRS	Risk of respiratory hospitalization	RR (2-day COH) = 1.04 (0.82–1.32) RR (3-day COH) = 0.95 (0.80–1.13)	RR (2-day COH) = 1.20 (1.00–1.43), by case crossover RR (3-day COH) = 1.15 (1.02–1.30), by time series

Sunyer et al. 2000	2,305 adults (≥ 35 years of age) Spain, 1985–1989	20-μg/m^3^ increase in same-day ambient black smoke	Respiratory mortality	OR = 1.14 (0.98–1.33)	OR = 1.52 (0.99, 2.31)

Sunyer et al. 2006	3,232 men and 3,592 women in Europe	Constant traffic density NO_2_ > 50 μg/m^3^	Prevalence of chronic phlegm	β (traffic) = 6.13% (4.37–8.32); *p*-trend = 0.47 β (NO_2_) = 6.67% (3.49–11.36); *p*-trend = 0.98	β (traffic) = 7.69% (5.95–9.75); *p*-trend = 0.002 β (NO_2_) = 8.68% (5.30–13.22); *p*-trend = 0.05

Thaller et al. 2008^a^	142 lifeguards 16–27 years of age (79% male)	10-μg/m^3^ increase daily average PM_2.5_, maximum O_3_	FVC FEV_1_/FVC	β (PM_2.5_) = −0.1% (−0.8 to 0.5) β (O_3_) = −0.006% (−0.2 to 0.05)	β (PM_2.5_) = −2.1% (−3.2 to −1.0) β (O_3_) = −0.3% (−0.4 to −0.6)

Studies reporting stronger effects among men					
Abbey et al. 1998	1,391 nonsmoking U.S. adults	IQR difference of 54.2 days/year > 100 μg/m^3^ PM_10_	PPFEV_1_ FEV_1_/FVC	β = −7.2 (−11.5 to −2.7) (males w/parental respiratory illness) β = −1.5 (−2.7 to −0.4)	β = 0.9 (−0.8 to 2.5) β = −0.2 (−0.9 to 0.5)

Galizia and Kinney 1999	520 nonsmoking undergraduate students in New Haven, CT	Lived ≥ 4 years in U.S. county with summer 1-hr O_3_ ≥ 80 ppb	Percent change in FEV_1_ FEF_25–75_ FEF_75_ symptoms	β = −4.7% (−0.7 to −8.8) β = −13.0% (−4.9 to −21.1) β = −10.0% (1.3 to −21.3) OR = 2.30 (1.15–3.46)	β = −0.26% (3.79 to −4.31) β = −1.96% (5.39 to −10.30) β = −2.08% (9.94 to −13.9) OR = 1.79 (0.83–3.89)

Korrick et al. 1998	530 hikers (18–64 years), Mt. Washington, NH	Ambient O_3_, PM_2.5_, aerosol acidity	Percent change in FEV_1_ FVC	β = −0.055 (SE = 0.025) β = −0.051 (SE = 0.016)	β = −0.039 (SE = 0.039) β = −0.019 (SE = 0.025)

Wang et al. 1999	1,075 Chinese adults (35–60 years)	Ambient PM_2.5_ and SO_2_ (rural vs. urban area)	Mean change FEV_1_	199 mL (SE = 50 mL)	87 mL (SE = 30 mL)

Studies reporting null or mixed modification					
Ackermann-Liebrich et al. 1997	9,651 adults 18–60 years of age in Switzerland (SAPALDIA cohort)	10-μg/m^3^ change in annual mean PM_10_	FVC	β = 3.4% (*p* < 0.05)^b^	Effects did not differ by sex

Chestnut et al. 1991	6,913 adults (25–75 years) (NHANES I)	1-SD increase in TSP (about 34 μg/m^3^)	Percent change in FVC	β = 2.25% (*p* < 0.05)	Effects did not differ by sex (*p* > 0.75)

Jedrychowski and Krzyzanowski 1989	584 men, 830 women in Krakow, Poland	Residence in area with higher sulfate or sulfur transformation ratio	Lung function, symptoms	FEV_1_ decline faster by 11 mL/year in high- vs. low-sulfate areas	High-sulfate area predicted symptoms, not lung function

Oosterlee et al. 1996	1,485 Haarlem, Netherlands, adults	Living on heavy (vs. light) trafficked streets	Wheeze (ever) Wheeze (2-year)	OR = 1.1 (0.8–1.3) OR = 1.1 (0.6–1.8)	Effects did not differ by sex

Zeka et al. 2006	1.9 million deaths in 20 U.S. cities, 1989–2000	10-μg/m^3^ change in daily PM_10_ concentrations	Percent increase in respiratory mortality	β = 0.71 (0.004–1.42)	β = 1.04 (0.33–1.75)

Abbreviations: IQR, interquartile range; NHANES, National Health and Nutrition Examination Survey; NR, not reported; OR, odds ratio; PPFEV_1_, percent predicted FEV_1_; RR, relative risk; Q, quartile; SAPALDIA, Study on Air Pollution and Lung Diseases in Adults; TRS, total reduced sulfur; VC%, vital capacity percent. Key results demonstrate observed effect modification, and are not exhaustive of results reported for each study. Values in parentheses are 95% confidence intervals, unless otherwise indicated.

a Other outcomes showed no significant effect modification by sex.

b Effects did not differ by sex, and therefore are reported here in only one column.

**Table 2:** Studies examining effect modification by sex among children.

Study	Population	Exposure metric(s)	Outcome(s) of interest	Risk among males	Risk among females
Studies reporting stronger effects among girls					
Brunekreef et al. 1997	877 Dutch children (7–12 years of age) in 1995	Truck traffic density (for children within 300 m of motorway)	Change in FVC FEV_1_	β = −1.1 (−6.7 to 4.9) β = −1.8 (−7.5 to 4.2)	β = −6.3 (−11.4 to −0.8) β = −6.2 (−11.5 to −0.6)

Luginaah et al. 2005	883 children (0–14 years of age) in Windsor, Ontario, Canada 1995–2000	IQR increase in 1-, 2-, 3-day lag NO_2_, SO_2_, CO, COH, O_3_, PM_10_, TRS	RR of respiratory hospitalization	RR(lag1 SO_2_) = 0.95 (0.87 to 1.04) RR(lag2 CO) = 0.996 (0.93 to 1.06) RR(lag2 CO) = 0.997 (0.87 to 1.14) RR(lag1 NO_2_) = 0.93 (0.81 to 1.07)	RR(lag1 SO_2_) = 1.11 (1.01 to 1.22) RR(lag2 CO) = 1.07 (1.00 to 1.14) RR(lag2 CO) = 1.19 (1.02 to 1.38) RR(lag1 NO_2_) = 1.19 (1.002 to 1.41)

Oftedal et al. 2008	2,307 9- and 10-year-old children in Oslo, Norway	IQR increase in lifetime NO_2_, PM_2.5_, PM_10_	Change in PEF FEF_25_ FEF_50_	β (NO_2_) = −69.1 mL/sec (−135.3 to −3.0) β (PM_10_) = −57.9 mL/sec (−116.2 to 0.4) β (PM_2.5_) = −30.1 mL/sec (−79.7 to 19.5)	β (NO_2_) = −94.5 mL/sec (−166.6 to −22.4) β (PM_10_) = −77.9 mL/sec (−141.9 to −14.0) β (PM_2.5_) = −68.9 mL/sec (−120.8 to −16.9)

Oosterlee et al. 1996	291 Haarlem, Netherlands, children (0–15 years of age)	Living on heavy (vs. light) trafficked streets	Wheeze (ever) Wheeze (1-year) Dyspnea (ever) Dyspnea (1-year)	OR = 1.2 (0.4–3.7) OR = 0.7 (0.2–2.5) OR = 0.9 (0.2–3.2) OR = 0.4 (0.1–2.6)	OR = 4.4 (1.4–13.6) OR = 5.3 (1.1–25.0) OR = 4.8 (1.3–17.7) OR = 15.8 (1.4–174.4)

Pershagen et al. 1995	197 children (4 months to 4 years) hospitalized with wheeze, 350 controls	Residential outdoor NO_2_, presence of gas stove	RR of wheezing bronchitis	RR (NO_2_ > 0.7) = 0.7 (0.4–1.3); *p*-trend = 0.10 RR (gas stove) = 0.9 (0.5–1.8)	RR (NO_2_ > 70) = 2.7 (1.1–6.8); *p*-trend= 0.02 RR (gas stove) = 2.4 (1.0–5.9)

Peters et al. 1999	3,293 children in 12 Southern California communities	Lifetime ambient NO_2_, PM_2.5_, and O_3_	FVC FEV_1_ PEFR MMEF	β (NO_2_) = −29.9 L/min (SE = 29.5) β (PM_2.5_) = 8.3 L/min (SE = 24.5) β (0_3_) = 52.0 L/min (SE = 65.8) β (PM_2.5_) = 32.0 L/min (SE = 30.1)	β (NO_2_) = −63.8 (SE = 18.3) β (PM_2.5_) = −47.6 (SE = 14.4) β (0_3_) = −250.9 (SE = 69.9) β (PM_2.5_) = −130.0 (SE = 30.3)

Rojas-Martinez et al. 2007	3,170 children (8 years of age) in Mexico City, 1996–1999	IQR increase in mean O_3_, PM_10_, NO_2_	Change in FEV_1_	β (O_3_) = −4 mL (−10 to 2) β (PM_10_) = −15 mL (−23 to −6) β (NO_2_) = −25 mL (−33 to −18)	β (O_3_) = −12 mL (−18 to −6) β (PM_10_) = −11 mL (−20 to −3) β (NO_2_) = −30 mL (−37 to −22)

Rosenlund et al. 2009	2,107 children 9–14 years of age in 40 Rome schools	Residential traffic Distance to busy road Modeled NO_2_	Percent difference in FEV_1_ FEF_25–75_	β = −4% (−29 to 21) β = −26% (−81 to 29)	β = −23% (−49 to 2); *p* for difference = 0.25 β = −103% (−163 to −43); *p* for difference = 0.06

Stern et al. 1989	1,630 children (7–12 years of age) in rural Canada	High- vs. low-exposure community	Percent difference in FVC FEV_1.0_	β = 1.45% (*p* < 0.05) β = 1.41% (*p* < 0.01)	β = 2.52% (*p* < 0.001) β = 2.03% (*p* < 0.001)

Van Vliet et al. 1997	1,498 children in 13 schools	Residence within 100 m of freeway	Chronic cough Wheeze	OR = 1.05 (0.50–2.22) OR = 1.29 (0.45–3.68)	OR = 2.45 (1.16–5.16) OR = 3.05 (1.11–8.41)

Studies reporting stronger effects among boys					
Delfino et al. 2004	14 boys and 5 girls with asthma, 9–17 years of age	IQR increase in 4-day personal PM_2.5_	FEV_1_	β = −16% (−26 to −6)	β = −1% (−16 to 14)

Gehring et al. 2002	1,756 German infants	Outdoor residential exposure gradient 1.5 μg/m^3^ in PM_2.5_, 0.4 × 10^−5^/m abs, 8.5 μg/m^3^ NO_2_	Cough without infection Dry cough at night	OR (PM_2.5_) = 1.43 (1.14–1.80) OR (abs) = 1.38 (1.11–1.71) OR (NO_2_) = 1.52 (1.16–2.00) OR (PM_2.5_) = 1.39 (1.08–1.78) OR (abs) = 1.31 (1.04–1.67) OR (NO_2_) = 1.45 (1.07–1.98)	OR (PM_2.5_) = 1.19 (0.84–1.70) OR (abs) = 1.25 (0.87–1.78) OR (NO_2_) = 1.22 (0.81–1.85) OR (PM_2.5_) = 1.17 (0.81–1.68) OR (abs) = 1.16 (0.79–1.71) OR (NO_2_) = 1.20 (0.78–1.84)

Jedrychowski et al. (1999)	1,001 children in Krakow, Poland	Residence in high- vs. low-pollution area	Slower growth in FVC FEV_1_	OR (FVC) = 2.15 (1.25–3.69) OR (FEV_1_) = 1.90 (1.12–3.25)	OR (FVC) = 1.50 (0.84–2.68) OR (FEV_1_) = 1.39 (0.78–2.44)

Peters et al. 1999	3,676 children in 12 Southern California communities	IQR difference in community lifetime ambient acid, NO_2_, PM_2.5_, O_3_	Prevalence of wheeze	OR (NO_2_) = 1.47 (1.04–2.09) OR (acid) = 1.55 (1.03–2.32)	OR (NO_2_) = 0.85 (0.59–1.21) OR (acid) = 1.08 (0.71–1.66)

Studies reporting null or mixed modification					
Emenius et al. 2003	540 Stockholm children (0–2 years of age)	Indoor and outdoor residential NO_2_	OR for recurrent wheeze (high vs. low quartile)	OR (outdoor NO_2_) = 1.60 (0.78–3.26)^a^ OR (indoor NO_2_) = 1.51 (0.81–2.82)	NR; effects did not differ by sex

Gauderman et al. 2004	1,759 children in 12 Southern California communities	Lifetime community annual average NO_2_, PM_2.5_, EC (most vs. least polluted)	Growth in FVC FEV_1_ MMEF	β (NO_2_) = −95.0 (−189.4 to −0.6)^a^ β (NO_2_) = −101.4 (−164.5 to −38.4) β (NO_2_) = −211.0 (−377.6 to −44.4)	NR; effects did not differ by sex

Lin et al. 2005	6,782 Toronto, Canada children, 0–14 years of age	6.5 μg/m^3^ increase in 6-day PM_10–2.5_ exposure	Hospitalizations for respiratory infections	β = 1.15% ( 1.02–1.30)	β = 1.18% (1.01–1.36)

Liu et al. 2009	182 asthmatic children 9–14 years of age in Windsor, Ontario, Canada	IQR change in same-day, lagged SO_2_, NO_2_, O_3_, PM_2.5_	Percent change in FEF_25–75_	β (same-day NO_2_ ) = −2.4 (−4.3 to −0.4) β (same-day PM_2.5_) = 1.9 (−3.5 to −0.3)	NR; effects did not differ by sex

Roemer et al. 1999	1,621 children in 14 European centers, 1993–1994	24-hr measures of PM_10_, BS, SO_2_, NO_2_	Change in evening PEF per 100 μg/m^3^	β (lag 0 SO_2_) = 1.9 L/min (*p* < 0.05) β (lag 0 BS) = 0.7 L/min (*p* < 0.10) β (lag 2 PM_2.5_) = −0.5 L/min (NS)	β (lag 0 SO_2_) = 1.4 (NS) β (lag 0 BS) = 0.2 (NS) β (lag 2 PM_2.5_) = 1.2 (*p* < 0.05)

Schwartz 1989	4,300 youths (6–24 years of age), NHANES II, 1976–1980	Annual average SO_2_, NO_2_, TSP, O_3_ at monitors	Change in FVC FEV_1_ PEF	β (NO_2_) = −2.94 (*p* = 0.0004) β (NO_2_) = −3.09 (*p* = 0.0003) β (NO_2_) = −3.23 (*p* = 0.0019)	NR; effects did not differ by sex

Smith et al. 2000	44 asthmatic children (< 14 years of age)	Daily personal NO_2_ exposure	Chest tightness	OR = 1.29 (1.16, 1.43)	NR; effects did not differ by sex

Zhao et al. 2008	1,993 pupils (11–15 years of age) in urban China	School indoor and outdoor SO_2_, NO_2_, O_3_	Asthma, wheeze	OR (wheeze, indoor SO_2_) = 1.18 (1.03–1.35)^a^ OR (wheeze, indoor CH_2_O) = 1.24 (1.03–1.48)	NR; effects did not differ by sex

Abbreviations: abs, absorbance; BS, black smoke; CH_2_O, formaldehyde; EC, elemental carbon; IQR, interquartile range; MMEF, median mid-expiratory flow; NR, not reported; NS, not significant; OR, odds ratio; PEFR, peak expiratory flow rate; RR, relative risk; TRS, total reduced sulfur. Key results demonstrate observed effect modification, and are not exhaustive of results reported for each study. Values in parentheses are 95% confidence interval, unless otherwise indicated.

a Effects did not differ by sex, and therefore are reported here in only one column.
